# WHO Systematic Assessment of Rehabilitation Situation (STARS): Results of the Field Testing in Jordan, Myanmar, Sri Lanka, Solomon Islands, Laos, Haiti, and Guyana

**DOI:** 10.3390/ijerph182111549

**Published:** 2021-11-03

**Authors:** Pauline Kleinitz, Carla Sabariego, Alarcos Cieza

**Affiliations:** 1 Department of Health Sciences and Medicine, University of Lucerne, 6002 Lucerne, Switzerland; carla.sabariego@paraplegie.ch; 2 Sensory Functions, Disability, and Rehabilitation Unit, Department for Noncommunicable Diseases, World Health Organization, 1211 Geneva 27, Switzerland; ciezaa@who.int; 3 Swiss Paraplegic Research, 6207 Nottwil, Switzerland; 4 Center for Rehabilitation in Global Health Systems, WHO Collaborating Center, University of Lucerne, 6002 Lucerne, Switzerland

**Keywords:** health system assessment, rehabilitation, global health

## Abstract

The WHO Systematic Assessment of Rehabilitation Situation (STARS) tool was developed by WHO to facilitate effective prioritization and strategic planning for rehabilitation in countries. The objective of this paper is to present the results of the fourth phase of its development, its field testing in seven countries focusing on its completeness, usefulness, accessibility and feasibility. Field testing occurred in Jordan, Myanmar, Sri Lanka, Solomon Islands, Laos, Haiti, and Guyana. Evaluation occurred through structured interviews and rating exercises with 17 government representatives, international consultants, WHO country or regional office staff and rehabilitation experts who were actively engaged and familiar with the STARS assessment and who were knowledgeable of the rehabilitation situation in the countries. STARS was appraised as relevant, complete and accurate in describing the country situation. Areas of inaccuracy were mostly linked to challenges in describing areas of services similarly when significant diversity existed. Feasibility and accessibility were mostly confirmed and more complex components of the tool as well as the guidance to the assessment process were slightly revised in light of the field-testing results. The field testing of WHO STARS confirmed its completeness, usefulness, accessibility and feasibility, and concerns raised by the interviews informed the last refinement of the tool. STARS is part of the WHO Rehabilitation in Health Systems-Guide for Action, available online, by September 2021, STARS had guided 21 country situation assessments.

## 1. Introduction

Recognizing the growth in rehabilitation needs in populations, yet the limited and under-resourced services that exist in many countries, the strengthening of rehabilitation is a growing priority [1]. Indeed, data from the Global Burden of Disease study shows that approximately 2.4 billion people currently have health conditions that could benefit from rehabilitation [2]. In response, the World Health Organization (WHO) launched the Rehabilitation 2030 Call for Action [3]. This initiative focuses on strengthening health systems to provide rehabilitation to anyone who needs it and ensuring availability of services at all levels and along the continuum of care.

Strengthening health systems to provide rehabilitation requires integration of rehabilitation into the components of a health system. The health system building block framework [4] helpfully characterizes these components; for example, to increase provision of rehabilitation services, rehabilitation should be integrated into health governance and services planning, health information systems, health financing and health workforce initiatives. Identifying what needs to be integrated is therefore one of the first steps to strengthening rehabilitation, and this requires an understanding of the situation so as to set priorities for action. To this end, a health system assessment (HSA) is a crucial step to comprehensively describe the situation and identify strengths, gaps and priorities, and the need for a tool that guides a comprehensive and well-structured assessment of rehabilitation was recognized.

A comprehensive and standardized HSA tool will facilitate more accurate assessments of the rehabilitation situation so as to identify priority areas for integration and action. Accordingly, a HSA tool shaped to rehabilitation was developed: The WHO Systematic Assessment of Rehabilitation Situation (STARS). The features of HSA tools were analyzed during the development of STARS and overwhelmingly they were tailored to the area of health that they assess, as this helps to guide a comprehensive assessment of the area (e.g., guide assessment of what is present in the country as well as what should be present). In this way they generate comprehensive findings that are important for informing prioritization. The tool is a key element and the first component of the WHO Rehabilitation in Health Systems-Guide for Action [5], a resource to support strategic planning for rehabilitation. STARS has three parts: a Manual which guides the assessment process in countries; a Template for Rehabilitation Information Collection (TRIC) which guides the information necessary for the assessment; and a Rehabilitation Maturity Model (RMM) which defines components for assessment and describes these on a 4-level maturity continuum.

STARS underwent a participatory development process with four phases; conceptualization; drafting; consultation and preliminary field test; and field testing, this process is outlined in Kleinitz et al. 2021 [6]. The objective of this paper is to present the results of the STARS field testing (fourth phase) carried out in Jordan, Myanmar, Sri Lanka, Solomon Islands, Laos, Haiti, and Guyana, whose goal was to test the completeness, usefulness, accessibility and feasibility of the tool.

## 2. Materials and Methods

This study received ethical approval from the WHO Ethics Committee.

The first three phases of the development of STARS resulted in the version of the tool that was ready for field testing and have been described in detail elsewhere (Kleinitz et al., 2021).

### 2.1. Systematic Assessment of Rehabilitation Situation Tool

The purpose of STARS is to facilitate an accurate assessment of the rehabilitation situation that will inform planning through identifying priority areas for integration and action. STARS has three parts, the first part is the Manual which guides the overall process, it is structured by the 4 steps for undertaking STARS in countries, these are; 1. Preparation; 2. Collection of data and information; 3. Conducting the assessment in the country, and 4. Report writing and dissemination. The Manual provides practical step-by-step guidance and includes samples of timelines, budgets, itineraries in countries, Rehabilitation Technical Working Group (RTWG) terms of reference and composition, frequently asked questions and a template for writing the report. The second component is the TRIC which guides information collection, it is structured by the building blocks, including a section on rehabilitation needs and emergency preparedness, it is designed to be sent to the Ministry of Health—Rehabilitation focal point approximately 8 weeks before the in-country assessment and completed by them, often aided by the country’s RTWG. The information collected is reported and informs the descriptions of the situation in the STARS report. The third component, the RMM defines the rehabilitation components for assessment and describes these across four levels on a maturity continuum, a system reflecting the ‘stages of growth’. The RMM (an excel document) is structured by integrating the health system building blocks and services outcomes along the logic model, this supports assessment of what capacity exists (inputs, e.g., workforce, expenditure), what services are available (outputs, e.g., rehabilitation at different levels of healthcare) and how these perform to achieve the desired outcomes, e.g., population coverage. The RMM is designed to be completed in the country with a consultant and the RTWG reflecting on where they are along the maturity continuum, the results (presented as tables and figures) are incorporated into the written report. The STARS development (Kleinitz et al. 2021) provides further detail regarding tool components. In this paper we are evaluating all three parts of STARS.

### 2.2. Selection of Countries and Assessors

A range in low- and middle-income counties (LMICs) representing as many WHO regions as possible, with large and small populations, a diversity of health system characteristics (e.g., large and small private sector), were targeted to pilot test STARS in a context as close as possible to the future scope of the tool. Nevertheless, the LMICs invited to participate in the STARS field testing represent a convenience sample of countries for which the Ministries of Health had previously engaged with WHO or a development partner on rehabilitation strengthening initiatives and demonstrated interest to utilize the STARS tool. Given the time and political commitment required by a Ministry of Health (MOH) to conduct comprehensive health systems assessments, the agreement and readiness of a MOH to conduct STARS was a core inclusion criterion in the selection of the countries.

STARS was implemented by consultants, selected based on their experience in rehabilitation and situation assessments, and trained in how to use STARS during a three-day workshop on the 13–15 June 2018, in Geneva. This training entailed presentations, small group work, case studies and simulations that facilitated familiarization, engagement with the tool and likely scenarios in countries. Consultants were not necessarily familiar with the countries where they utilized the STARS. Therefore, we ensured a structured preparation phase including reviewing the information within the TRIC, and then in-country site visits, interviews and discussions to familiarize them with the country situation.

### 2.3. Evaluation of Completeness, Usefulness, Accessibility and Feasibility

The evaluation entailed brief structured interviews with key individuals who met the twin criteria of being actively engaged and familiar with the STARS assessment and having direct knowledge of the rehabilitation situation in the country. For recruiting the participants, invitations were emailed to the international consultants who conducted the STARS, ministry of health rehabilitation focal points and other rehabilitation experts living in or working with the selected countries, and relevant WHO country and/or regional office staff.

Interviews included open-ended questions with a corresponding rating exercise (Table 1), and respondents were also asked to rate each of the components of the RMM in relation to their importance in a 4-level scale ranging from not important to very important. Interviewees received it in advance per e-mail. Responses prior to the interview were encouraged and when interview time was limited, further written responses were provided in follow up. Interviews were conducted by (PK) either over the phone or face-to-face. Open-ended questions were transcribed verbatim.

### 2.4. Data Analyses

Key information from open-ended answers and comments was extracted and consolidated into a single document focusing on the core points of each question (Table 1). The analysis then occurred in three stages: coding text, development of descriptive themes and generation of analytical themes (interpretation). Since the structure of the questions were related to the characteristics sought (e.g., completeness), the components of STARS (Manual, TRIC, RMM) and the process of its utilization in countries (how well it worked in countries), these became the first line of coding with subsequent codes assigned during the reading. The subsequent codes assigned tended to the revisions that could improve STARS. This had the advantage of allowing synthesis of information in a succinct manner that could directly inform revisions to the actual product. Analysis included looking for overall trends and trends stratified by interviewee group or country. Where helpful to illustrate common feedback, quotations were coded.

Descriptive statistics (mean, median, range, absolute numbers and percentages) were estimated for the 5 key informant questions, and the mean, median, absolute numbers and percentages were calculated for the rating scales of the RMM components, all using Microsoft Excel.

## 3. Results

The field testing occurred over a 20-month period, from July 2018 to March 2020. Selected countries included Jordan, Myanmar, Sri Lanka, Solomon Islands, Laos, Haiti and Guyana. They represent four WHO regions, include LMICs, countries with large and small populations, and health systems with large and small proportions of non-government providers. Altogether 21 invitations were sent out and 17 structured interviews conducted. The RMM rating was undertaken by 16 people, as one interviewee from a WHO country office declined the task due to being unfamiliar with rehabilitation. In this case, 12 interviews were conducted over the telephone and five face-to-face, two of the interviewees were WHO Regional Advisors that shared their experience across two countries in their regions (America’s and Western Pacific regions). See Table 2.

### 3.1. Evaluation of Completeness, Usefulness, Accessibility and Feasibility

#### 3.1.1. Completeness

Completeness explored to what extent STARS covers all aspects relevant to rehabilitation. Out of all aspects of STARS, its completeness scored most positively: 100% of interviewees scoring it eight or higher, with a mean of 8.88 and median of 9 and score range from 8–10 (Table 3). In the open-ended questions, thematic analysis revealed that most interviewees considered that STARS had high levels of completeness and relevance of assessment components. In this case, 10 persons reported areas missing, these included: history of the sector, and rehabilitation for vision, hearing and mental health conditions. Seven interviewees answered that nothing was missing.

#### 3.1.2. Usefulness

Usefulness explored to what extent STARS provided an accurate picture of rehabilitation in the country. 76% of interviewees rated usefulness positively with a score of eight or higher, the mean was 8.12, the median was 8 the score range was 5–10. Areas of inaccuracy or imprecision that were reported included descriptions of private sector rehabilitation provision, geographic differences within a country and quality of rehabilitation. Five interviewees (three consultants and two WHO staff) reported challenges when undertaking STARS to progress beyond ‘particular people’s perspectives’ and past ‘gatekeepers’ to describe the ‘real’ situation.

#### 3.1.3. Accessibility

Accessibility assessed the extent to which STARS was considered easy to implement and complete.

STARS Manual: 16 out of 17 interviewees scored accessibility of the Manual moderately high (seven or more), meaning it was relatively easy to use. The mean was 7.76, the median was 7 and the score range for this was 6–10. The interviewees welcomed the Manual’s step by step and practical guidance. Seven of the interviewees did not identify any areas for improvement. Suggestions for improvement included the need for the Manual to be translated to country languages, the need for government familiarization and training on the manual and creation of sub-steps.

STARS Template for information collection: 13 out of 17 interviewees scored accessibility moderately high (seven or more). The mean was 7.12, the median was 7 and the score range was from 2–9. A concern was that the template asks for information that many governments could not provide, e.g., rehabilitation expenditure. The government stakeholders in three countries misunderstood the instructions and requested multiple stakeholders to complete it, which then required consolidation. Suggestions for its improvement focused on strengthening the guidance for how it should be completed.

STARS Rehabilitation Maturity Model. 11 out of 17 interviewees scored accessibility of this moderately high (seven or more). The mean was 6.47, the median was 7 and score range was from 1–8. In comparison to the other parts, over 50% of interviewees characterized the RMM as ‘complex’. Some components were considered difficult to score for being “subjective” and it required more time with government to complete than what had been indicated in the guidance. Suggestions for improvement related to: reducing component numbers; removing use of sub-components; clearer differentiation between maturity levels; translation to local languages; training of on its purpose and how to complete it; and managing governments expectations about likely scores and their interpretation.

#### 3.1.4. Feasibility

14 out of 17 interviewees scored feasibility as moderately high (seven or more). The mean was 7.35, the median 8 and score range was 3–10. Two areas of concern were mentioned. First, the length of time it took to complete the assessment in a country, suggesting that the recommended two weeks was not long enough for larger and/or more complex countries. Second, the necessity of additional partners, such as the WHO country office and an international non-government organization, to be engaged and supporting government through the process.

#### 3.1.5. Rating the Importance of the Rehabilitation Maturity Model Components

The mean score was calculated for each component and 14 out of 56 scored 3.5 or less, the remaining 42 components had a mean above 3.5 (Table 4). The 14 components with a mean of 3.5 or less included: capacity and levers for rehabilitation plan implementation; rehabilitation transparency; rehabilitation regulation; assistive technology legislation, policies and plans; information generated on rehabilitation needs in the population; rehabilitation in hospital, community and education settings for children with delays and disabilities; rehabilitation for targeted population health needs; rehabilitation effective and efficient dosages; equitable across the functioning spectrum; and technical and allocative efficiency.

Analysis of data included looking for trends across stratified groups, either across countries or stakeholder groups, this was accomplished by comparing the means and with a small sample size the analysis did not reveal any significant trends.

Interviews revealed commonalities regarding the positive features and concerns regarding specific components of STARS, as well as exploring reasons for these and considerations of what could be carried out to improve the tool. Bringing stakeholders together in countries through RTWGs mostly worked well. It increased the quality of input and amount of information shared, contributing to greater insights regarding the country situation, as reported for five countries (Guyana, Myanmar, Haiti, Sri Lanka and Solomon Islands). In Laos and Jordan working groups were considered less effective at generating information and insights from development partners was valued, particularly by consultants.

The comprehensive guidance of the RMM was appreciated and its structure supported the development of conclusions related to overall access and outcomes of rehabilitation, that help to ‘tell the story’ of what rehabilitation had achieved, or not. The consultants valued the tool’s prompts to consider overall outcomes such as service coverage, sustainability, and equity. However, they also reported that this section of the report was difficult to elaborate with limited data to draw upon and therefore the conclusions had to be cautiously made. The visual scoring was considered valuable and facilitated quicker comprehension of results.

Interviews explored the reasons given for the concerns raised about areas of inaccuracy or imprecision related to descriptions of the private sector rehabilitation provision, these were described similarly and related to the limited access to the private sector services during the in-country assessment period due to the MOH focus on government services and few private services visited. The reason for concerns about difficulty to describe the situation across geographic areas was explored and reported to be because the 2-week period in a country prevented visits beyond major cities and a small selection of rural areas. The reasons for concerns about describing quality of services were reported to be because of significant variations across facilities and the need to rely on observation and stakeholder reporting rather than any objectives measures which were mostly unavailable.

Finally, comments directed towards what could have been carried out differently during the STARS process in the country yielded similar responses to the questions focused on what improvements could be made to the tools. However, the need for engagement from senior government officials during the process and the value of WHOs role to support government prioritization of rehabilitation was also stressed.

### 3.2. STARS Revision by WHO

Revision to the STARS Manual focused on creating more clearly defined sub-steps under each step and expanding guidance for the preparatory phase. For example, preparatory guidance was adjusted to include development of a detailed concept note prior to commencement, and more instructions for how to use the RMM with governments. Small revisions were made to the information collection template and its name was changed to reduce the misunderstanding that multiple people should complete it, a note was added that limited available data for some questions is common and need not be a concern to governments. The RMM underwent changes including explicitly mentioning rehabilitation for people with hearing, vision and mental health needs, refining definitions of components, increasing differentiation and uniformity in maturity level descriptions through consistent use of the terms ‘emerging, minimal, moderate and high’, and reducing the number of components. The RMM now has 50 components with revised overall maturity descriptions. The STARS is available on the WHO website.

## 4. Discussion

We present results of the WHO STARS field testing in Jordan, Myanmar, Sri Lanka, Solomon Islands, Laos, Haiti, and Guyana. The interviews conducted targeted the completeness, usefulness, accessibility and feasibility of STARS. Results confirmed that STARS can effectively guide an HSA to accurately reflect the rehabilitation situation in countries and that this best occurs through collaboration between the government, consultants, rehabilitation experts, and development partners, including WHO. Concerns raised in the interviews guided the last revision of STARS, now available as part of the WHO Rehabilitation in Health Systems-Guide for Action [5].

Participatory approaches in the development of HSA tools are important to achieve relevant, accessible, and feasible tools that governments want to use [7]. Key stakeholders, such as the Universal Health Coverage 2030 Technical Working Group for HSAs, have emphasized the value of demand driven tools [8]. Prominent HSA tools, such as the Joint Assessment of National Strategies [9] and the Health System Assessment Approach: A How-To Manual [10], have been developed using participatory approaches with end user input informing initial and revised versions. For this reason, while expert and user input was crucial during the first three phases of the development of STARS, the user input was prioritized during the field-testing. Field testing has been used for development of similar tools, for example one that assesses HIV-related stigma among health facility staff [11] and one that measures primary healthcare performance [12]. It was also noted that some HSAs did not undergo field testing (or at least publish their field testing), but they did have multiple versions over time that indicate that field testing is a useful phase of HSA development. The positive appraisal of STARS observed in the field testing suggests the participatory approach was successful. Nevertheless, raised concerns were key for relatively minor but important refinements. By September 2021, STARS had guided 21 country situation assessments.

The Rehabilitation 2030 Call for Action stresses the importance of integrating rehabilitation into all health system components, therefore a tool reflecting this breadth was necessary, and high levels of completeness were sought during STARS development. The breadth of information is important as studies have highlighted the diversity of issues in countries, such as inadequate rehabilitation workforce [13], and gaps in the data collected through heath information systems [14]. The challenge faced in developing a HSA lies in making assessments and corresponding reports ‘complete enough’ while still being feasible in terms of their length and completion time. The completeness of STARS was considered high, reflected in both the rating of STARS reports and the individual RMM components. Moreover, the field testing mostly confirmed an appropriate balance between completeness and feasibility, with exception of the RMM, which was reduced in the revision from 56 to 50 components.

The ability of a tool to provide an accurate picture of a country situation is crucial, especially as its findings are used during planning to determine which of multiple priorities requires greater attention. Interviewees considered STARS reports accurate, with two exceptions. The first area reported was an inaccuracy in the descriptors used for particular components of rehabilitation services (e.g., private sector, services across geographic areas). When explored, this inaccuracy related more to an ‘inadequacy’ or imprecision in descriptions, reports overly characterized services similarly in an attempt to keep descriptions brief. Linked to inaccuracy, the challenge of describing the ‘real’ situation including politics and power hierarchies was also reported. This challenge is not specific to STARS and innate in most HSA processes, and often descriptions of the politics and power hierarchies are intentionally omitted due to their sensitive and sometimes subjective nature. Both of these areas highlight the challenge of all HSAs as they try to achieve a balance of features, for example being accurate and complete but also remaining succinct and sensitive to differing local perspectives.

Many HSAs tools have incorporated grading systems with some of these characterized as maturity models. Maturity models, or ‘stages of growth’, are based on the premise that people, organizations, programs move through a process of development towards a more advanced state and that this occurs over multiple levels [15]. In healthcare they are increasingly utilized for assessment of technology, health information systems, quality, hospital management [16] as well as across program areas such as immunization [17]. These exercises can serve two key purposes: engagement of stakeholders in the assessment process facilitating their reflection on where they are now and the pathway forward, and the support of a quicker, more reproducible analysis that allows for presentation of findings in an accessible format. The initial decision to develop the RMM was based on this purpose but was described in the field testing as complex and overwhelming on first use. As government stakeholders will mostly use it once, accessibility concerns were addressed. In addition, individuals working regionally, for instance, WHO regional officers, and researchers have queried if the RMM could be used for cross-country comparisons and to track change over time. Comparability was not a primary objective of the RMM, nor is it often an objective of HSAs [18], however having some elements of STARS as comparable was considered important. This was achieved through the TRIC, which collects well-defined comparable data that can be used to track changes over time in a country and to expand the evidence on the global availability of rehabilitation services and therefore to plan regional support. The information collected through TRIC and reported in the STARS reports is now being used for a cross country comparison study and is the focus of a forthcoming paper.

The added value of STARS is its ability to generate comprehensive overviews to the rehabilitation situation in many LMICs, these provide decision makers with evidence and insights into their situation and improve their national rehabilitation planning efforts. For all of the seven countries included in this study, conducting STARS was the first comprehensive rehabilitation situation assessment they had undertaken. Indeed, few comprehensive rehabilitation situation assessments have been published and governments have often made planning decisions with limited data mostly because rehabilitation hasn’t been well integrated into health information systems making data unavailable [19]. STARS demonstrates the type of information and data relevant for monitoring progress in countries and in this way increases recognition of its value and feasibility, STARS has the potential to positively impact on rehabilitation in many LMICs.

Limitations. Our study must be interpreted in light of its limitations. Although a broad range of diverse countries were included in the field-testing, and STARS was positively evaluated, different results might be obtained in other settings, and challenges were observed for settings in which the population is large and the health system more complex. Moreover, we aimed to check completeness, usefulness, accessibility and feasibility, but we are aware that formally checking those characteristics would require a more systematic approach. For instance, confirming completeness could involve a systematic review of rehabilitation features and a structured comparison with STARS. Nevertheless, the approach used in the field testing was sufficient to fulfill our goal of generating a final version of STARS. Future utilization therefore should include further evaluations of its strengths and weaknesses and revisions to STARS should be anticipated.

## 5. Conclusions

The field testing of WHO STARS in seven countries broadly confirmed its completeness, usefulness, accessibility and feasibility, and concerns raised by the interviews with rehabilitation experts (government and non-government), international consultants and WHO staff informed the last refinement of the tool. STARS is part of the WHO Rehabilitation in Health Systems-Guide for Action, available online. By September 2021, STARS had guided 21 country situation assessments and reports have been successfully used to inform rehabilitation strategic planning and integration into other national health planning processes. In this way, situation assessments conducted with STARS became a crucial element to strengthening rehabilitation in health systems, as demanded in the Rehabilitation 2030 Call for Action.

## Figures and Tables

**Table 1:** Key Informant Interview questions for examination of completeness, usefulness, accessibility and the feasibility of STARS.

Characteristic	Interview Questions										
Completeness	To what extent did the tool cover all aspects that are relevant to rehabilitation?										
	No aspects relevant to rehabilitation All aspects relevant to rehabilitation										
	0	1	2	3	4	5	6	7	8	9	10
	What areas were missing?										
Usefulness	To what extent did the tool provide an accurate picture of rehabilitation in a country?										
	Not accurate picture of rehabilitation Accurate picture of rehabilitation										
	0	1	2	3	4	5	6	7	8	9	10
	What were the areas of inaccuracy?										
Accessibility	To what extent was the tool easy to implement and be completed? These questions were repeated for each of the three STARS components, the STARS Manual, Rehabilitation Capacity Questionnaire and Rehabilitation Maturity Model.										
	Not easy to implement and complete Easy to implement and complete										
	0	1	2	3	4	5	6	7	8	9	10
	Explain why the STARS Manual/Rehabilitation Capacity Questionnaire/Rehabilitation Maturity Model was not easy to implement or complete?										
	What suggestions do you have to improve this?										
Feasibility	To what extent was the tool feasible to be implemented? When answering this question please consider the feasibility in terms of commonly applied parameters such as, number of days to complete, associated cost, information availability and time to retrieve it, availability of key informants in-country, and ability of WHO staff to support assessments while in-country.										
	Not feasible at all Feasible										
	0	1	2	3	4	5	6	7	8	9	10
	Explain what aspects of the tool are not feasible and what suggestions do you have to improve this?										
Overall comments	What worked well? What did not work well? What would you do differently?										

**Table 2:** Composition of Interviewees.

Country STARS Occurred within	Government Representative	National Rehabilitation Experts	Consultant	WHO Country or Regional Staff
GUYANA	1	-	1	1 (WHO Americas)
JORDAN	1	1	1	1 (Country staff)
MYANMAR	1	1	-	1 (Country staff)
SRI LANKA	1	-	-	1 (Country staff)
HAITI	-	-	1	1 (WHO Americas)
SOLOMON ISLANDS	1	1	-	1 (WHO Western Pacific)
LAOS	-	-	1	1 (WHO Western Pacific)

**Table 3:** Key Informant Interview—Results of Rating Exercise.

Key Informant Interview Question	Total Respondents	Score on Scale from 1 (Lowest/Negative) to 10 (Highest/Positive) Number of Respondents for Each Score										Mean	Median	Range
		1	2	3	4	5	6	7	8	9	10			
1. To what extent did the tool cover elements relevant to rehabilitation?	17	0	0	0	0	0	0	0	7	5	5	8.88	9	8–10
2. To what extent did the tool provide an accurate picture of rehabilitation in the country?	17	0	0	0	0	1	1	2	7	3	3	8.12	8	5–10
3a. To what extent was the tools STARS Manual easy to implement and be completed?	17	0	0	0	0	0	1	9	2	3	2	7.76	7	6–10
3b. To what extent was the tools TRIC easy to implement and be completed?	17	0	1	0	0	0	3	5	6	2	0	7.12	7	2–9
3c. To what extent was the tools RMM easy to implement and be completed?	17	1	0	0	0	2	3	7	4	0	0	6.47	7	1–8
4. To what extent was the tool feasible to be implemented?	17	0	0	1	0	2	0	3	9	1	1	7.35	8	3–10

**Table 4:** Rehabilitation Maturity Model Components—Results of Rating Exercise.

Component of the Rehabilitation Maturity Model	Total Respondents	Score across 4 Levels of Importance % of Respondents for Each Level				Mean	Median	Range
		Not Important (Score-1)	A Little Important (Score-2)	Somewhat Important (Score-3)	Very Important (Score-4)			
GOVERNANCE								
1. Rehabilitation has legislation, policies and plans	16	0%	0%	12.5%	87.5%	3.88	4	3–4
2. There is high level coordination for rehabilitation	16	0%	0%	31%	69%	3.69	4	3–4
3. There is planning to expand the service delivery of rehabilitation	16	0%	0%	12.5%	87.5%	3.88	4	3–4
4. The capacity and levers for rehabilitation plan implementation are in place	16	0%	0%	50%	50%	3.50	3.5	3–4
5. There is high level accountability and reporting for rehabilitation	16	0%	0%	25%	75%	3.75	4	3–4
6. There is transparency for rehabilitation	16	0%	12.5%	50%	37.5%	3.25	3	2–4
7. Rehabilitation is regulated	16	0%	12%	44%	44%	3.31	3	2–4
8. There is leadership, collaboration and coalition building for rehabilitation	16	0%	0%	12.5%	87.5%	3.88	4	3–4
9. Assistive Technology has legislation, policies and plans	16	6%	0%	31%	63%	3.50	4	1–4
10. Assistive Technology has effective AT procurement	16	0%	6%	31%	63%	3.56	4	2–4
INFORMATION								
11. Information is generated about rehabilitation needs, including population functioning and disability	16	0%	6%	44%	50%	3.44	3.5	2–4
12. Information is generated about the availability and utilization of rehabilitation services	16	0%	0%	6%	94%	3.94	4	3–4
13. Information is generated about outcomes, quality and efficiency of rehabilitation services	16	0%	0%	37.5%	62.5%	3.63	4	3–4
14. Information is used to inform policy and programme decision making	16	0%	0%	25%	75%	3.75	4	3–4
FINANCING								
15. The financing for rehabilitation covers all the population	16	0%	0%	19%	81%	3.81	4	3–4
16. The financing for rehabilitation covers a wide range of prioritized services	16	0%	0%	19%	81%	3.81	4	3–4
17. The financing for rehabilitation prevents financial hardship	16	0%	6%	25%	69%	3.63	4	2–4
WORKFORCE								
18. There is adequate workforce available, it is sustainable and aligned to the market needs	16	0%	0%	12.5%	87.5	3.88	4	3–4
19. The workforce is trained with appropriate skills to match tasks and meet need	16	0%	0%	6%	94%	3.94	4	3–4
20. The rehabilitation workforce is well managed and planned	16	0%	0%	12.5%	87.5%	3.88	4	3–4
21. The rehabilitation workforce is motivated and supported	16	0%	0%	31%	69%	3.69	4	3–4
SERVICE ACCESSIBILITY—AVAILABILITY/AFFORDABILITY/ACCEPTABILITY								
22. Rehabilitation is available across tertiary levels of health care	16	0%	0%	6%	94%	3.94	4	3–4
23. Rehabilitation is available across secondary levels of health care	16	0%	0%	6%	94%	3.94	4	3–4
24. Rehabilitation is available in primary healthcare	16	0%	6%	19%	75%	3.69	4	2–4
25. Rehabilitation is delivered in community settings	16	0%	6%	13%	81%	3.75	4	2–4
26. Rehabilitation is available across the acute phases of care	16	0%	0%	31%	69%	3.69	4	3–4
27. Rehabilitation is available across the sub-acute phases of care	16	0%	0%	31%	69%	3.69	4	3–4
28. Rehabilitation is available across the long-term phases of care	16	0%	0%	31%	69%	3.69	4	3–4
29. Assistive Products are available	16	0%	0%	6%	94%	3.94	4	3–4
30. Assistive Product follow-up and maintenance is available	16	0%	0%	12.5%	87.5%	3.88	4	3–4
31. Rehabilitation is available for adults with complex rehabilitation needs	16	0%	0%	31%	69%	3.69	4	3–4
32. There is early identification and referral to rehabilitation for children with developmental delays and disabilities	16	0%	0%	31%	69%	3.69	4	3–4
33. There is rehabilitation available in hospital and clinical settings for children with developmental delays and disabilities	16	0%	0%	50%	50%	3.50	3.5	3–4
34. There is rehabilitation available in community settings during early childhood for children with developmental delays and disabilities	16	0%	6%	50%	44%	3.38	3	2–4
35. There is rehabilitation available in community settings during school age for children with developmental delays and disabilities	16	0%	19%	56%	25%	3.06	3	2–4
36. Rehabilitation is available for target populations in need	16	0%	13%	81%	6%	2.94	3	2–4
37. Rehabilitation infrastructure, equipment and medicines are available	16	0%	0%	12.5%	87.5%	3.88	4	3–4
SERVICE QUALITY								
38. Rehabilitation is effective, it utilizes evidence-based interventions	16	0%	6%	19%	75%	3.69	4	2–4
39. Rehabilitation is effective, it utilizes effective and efficient dosages of rehabilitation interventions	16	0%	12.5%	25%	62.5%	3.50	4	2–4
40. Rehabilitation is effective, it is timely and delivered along a continuum of care	16	0%	0%	31%	69%	3.69	4	3–4
41. Rehabilitation is person-centered, it empowers and engages users, family, carers	16	0%	0%	31%	69%	3.69	4	3–4
42. Rehabilitation is convenient, and socially and culturally acceptable	16	0%	0%	37.5%	62.5%	3.63	4	3–4
43. Rehabilitation is safe	16	0%	0%	25%	75%	3.75	4	3–4
OUTCOMES AND ATTRIBUTES								
44. Rehabilitation is Accessible—it is available to all who need it	16	0%	0	19	81	3.81	4	2–4
45. Rehabilitation is Accessible—it is affordable to all who need it	16	0%	0	19	81	3.81	4	2–4
46. Rehabilitation is Accessible—it is acceptable to all who need it	16	0%	6	38	56	3.50	4	2–4
47. Rehabilitation is Equitable—across spectrum of functioning in the population	16	0%	12	44	44	3.31	3	2–4
48. Rehabilitation is Equitable—across disadvantaged population groups	16	0%	6	19	75	3.69	4	2–4
49. Rehabilitation is Efficient—it has allocative efficiency	16	0%	13	56	31	3.19	3	2–4
50. Rehabilitation is Efficient—it has technical efficiency	16	0%	6	63	31	3.25	3	2–4
51. Rehabilitation is Accountable—Governing Agencies are accountable	16	0%	6	31	63	3.56	4	2–4
52. Rehabilitation is Accountable—Service Providers are accountable	16	0%	6	31	63	3.56	4	2–4
53. Rehabilitation is Accountable—Practitioners are accountable	16	0%	6	31	63	3.56	4	2–4
54. Rehabilitation is Sustainable—it has financial sustainability	16	0%	6	19	75	3.69	4	2–4
55. Rehabilitation is Sustainable—it has institutional sustainability	16	0%	6	19	75	3.69	4	2–4
56. Rehabilitation is Sustainable—it is resilient to crisis and disaster	16	0%	6	31	63	3.56	4	2–4

## Data Availability

Data is contained within the article.
